# Authentication of a novel antibody to zebrafish collagen type XI alpha 1 chain (Col11a1a)

**DOI:** 10.1186/s13104-021-05770-x

**Published:** 2021-09-15

**Authors:** Jonathon C. Reeck, Makenna J. Hardy, Xinzhu Pu, Cynthia Keller-Peck, Julia Thom Oxford

**Affiliations:** 1 Department of Biological Sciences, Biomolecular Sciences Graduate Program, and Biomolecular Research Center, Boise State University, Boise, ID 83725 USA; 2 Biomolecular Sciences Graduate Program, Biomolecular Research Center, Boise State University, Boise, ID 83725 USA; 3 Biomolecular Research Center, Boise State University, Boise, ID 83725 USA

**Keywords:** Collagen α1(XI), Col11a1a, Cartilage, Zebrafish, Antibody authentication, Immunoblot, Immunofluorescence microscopy

## Abstract

**Objective:**

Extracellular matrix proteins play important roles in embryonic development and antibodies that specifically detect these proteins are essential to understanding their function. The zebrafish embryo is a popular model for vertebrate development but suffers from a dearth of authenticated antibody reagents for research. Here, we describe a novel antibody designed to detect the minor fibrillar collagen chain Col11a1a in zebrafish (AB strain).

**Results:**

The Col11a1a antibody was raised in rabbit against a peptide comprising a unique sequence within the zebrafish Col11a1a gene product. The antibody was affinity-purified and characterized by ELISA. The antibody is effective for immunoblot and immunohistochemistry applications. Protein bands identified by immunoblot were confirmed by mass spectrometry and sensitivity to collagenase. Col11a1a knockout zebrafish were used to confirm specificity of the antibody. The Col11a1a antibody labeled cartilaginous structures within the developing jaw, consistent with previously characterized Col11a1 antibodies in other species. Col11a1a within formalin-fixed paraffin-embedded zebrafish were recognized by the antibody. The antibodies and the approaches described here will help to address the lack of well-defined antibody reagents in zebrafish research.

**Supplementary Information:**

The online version contains supplementary material available at 10.1186/s13104-021-05770-x.

## Introduction

Extracellular matrix (ECM) plays key roles during embryonic development [1–5], and the minor fibrillar collagens play regulatory roles in collagen assembly and structural integrity of tissues [6–11]. While in situ hybridization demonstrates location of mRNA, it does not always indicate the location of the resulting protein [6, 8], particularly for secreted ECM proteins with long half-lives.

Collagen type XI is a trimeric molecule consisting of α1, α2, and α3 chains. The α1(XI) and α2(XI) chains possess unique amino terminal domains (NTD) that contain amino propeptides and variable regions, that are retained at the surface of collagen fibrils for extended periods of time. Collagens persist in tissues [12, 13], and undergo modifications such as alternative splicing of the primary transcript, proteolytic cleavage of the procollagen, glycosylation and crosslinking [14, 15] adding to complexity. In this case, antibodies are essential tools to monitor spatiotemporal changes [12, 16].

The structure of collagens presents a challenge for accessing epitopes for immunolocalization. Sequence conservation among collagens increases the challenge associated with the design and development of specific antibodies for research [17, 18]. The NTD of minor fibrillar collagens offers attractive targets for specific protein recognition [11, 16, 19, 20]. The location and slow proteolytic processing make the NTD a suitable epitope target for antibody-based detection.

While zebrafish offer many advantages, including optical transparency and *ex utero* development [21, 22], one limitation is the paucity of antibodies for zebrafish ECM research. We describe antigen selection and antibody development of a novel Col11a1a antibody. Antibody validation is critical in research [23–25].

## Main text

### Methods

#### Zebrafish husbandry

This study was performed under Boise State University (AC18-014 and AC18-15). Zebrafish (AB, Zebrafish International Resource Center (ZIRC Eugene, Oregon, USA; zebrafish.org) were housed under standard conditions [26]. Developmental staging was reported as hours post-fertilization (hpf) at 28.5 °C. Embryos were raised to 24–72 hpf in egg water (pH 7.2) [26]. Homozygous CRISPR/Cas9-generated knockout of Col11a1a was lethal in the majority of offspring [18]; therefore, heterozygous crosses were used to generate embryos to validate antibody. Embryos were humanely euthanized at 24–72 hpf on ice for 30 min followed by tricaine for 10 min, frozen, then fixed in 4% paraformaldehyde (PFA). No animals were excluded. Potential confounders were not controlled. A total of 105 embryos were used, the minimum number to allow detection of protein.

#### Antibody design and development

Antibodies were generated using the peptide sequence NH_2_-ck(g)_9_dvphkdtlqa-COOH conjugated to keyhole limpet hemocyanin. Custom primary antibody production was outsourced to Bethyl Laboratories, Inc., Montgomery, Texas USA (bethyl.com). Rabbits were immunized and sera were collected. Sequential bleeds were screened by ELISA against the peptide to determine titer. Rabbits were euthanized according to ethical use protocols held by Bethyl Laboratories. Antibodies were affinity purified, concentrated to 5 mg/mL, and stored at − 20 °C.

#### Protein isolation and detection by immunoblot

Wildtype, heterozygotes (Col11a1a^+/−^) and homozygotes (Col11a1a^−/−^) embryos were used for protein isolation and detection. Experimental groups contained 20 embryos. Embryos were dechorionated using 1 mg/mL pronase at room temperature then rinsed in Ringer’s solution. Embryos were treated with ethylenediamine tetraacetic acid and protease/phosphatase inhibitor cocktail (Thermo Fisher Scientific) in Ringer’s and passed through a glass pipette to remove the yolk.

Samples were processed as previously described [27] with modification. In brief, samples previously fixed in 4% paraformaldehyde (PFA) were incubated in 2% sodium dodecyl sulfate (SDS) at 95 °C for 30 min and 60 °C for 2 h to reverse PFA fixation. The embryos were centrifuged for 20 min at 5000×*g* and the supernatant was removed. De-yolked embryos were homogenized in SDS sample buffer with a microfuge pestle, boiled, centrifuged to clarify, and proteins were separated on a 4–12% gradient bis–Tris gel. After transfer to polyvinylidene difluoride membranes, they were blocked for 60 min at room temperature. Membranes were rinsed with Tween-20 Tris buffered saline (TTBS) 3 × 5 min. Primary antibody was added to the membrane at a 1:1000 dilution in TTBS with 5% bovine serum albumin and incubated overnight at 4 °C with constant rocking. For peptide blocking experiments, the peptide was added to the primary antibody solution and agitated for 1 h before adding to the membrane. The primary antibody solution was decanted, and unbound antibody was removed by rinsing the membrane with TTBS 3 × 5 min. Secondary antibody conjugated to horseradish peroxidase was added to the membrane and rocked at room temperature for one hour. The secondary antibody was decanted, and the membrane washed 3 × 5 min with TTBS. Enhanced chemiluminescence reagent was added to the membrane. Imaging and quantification was performed on a FluorChem E Digital Darkroom. Mean intensity plus and minus standard deviation was reported, N = 3 independent replicates.

#### Liquid chromatography–tandem mass spectrometry (LC–MS/MS)-based protein sequence analysis

LC–MS/MS analysis was performed using methods established previously [28] with modifications. Briefly, excised gel pieces were destained in 50% acetonitrile and 50 mM NH_4_HCO_3_, followed by the treatment with dithiothreitol (10 mM) to reduce disulfide bonds and iodoacetamide (55 mM) for alkylation and then digested with proteomics grade trypsin (Thermo Fisher Scientific, Waltham, MA, USA) overnight at 37 °C. Peptides were extracted, dried under vacuum, and reconstituted in 5% acetonitrile and 0.1% formic acid. Tryptic peptides were analyzed on a Velos Pro Dual-Pressure Linear Ion Trap mass spectrometer equipped with a nano electrospray ionization source and coupled with an Easy-nLC II nano LC system (Thermo Fisher Scientific).

Peptide spectral matching and protein identification were achieved by database search using Sequest HT algorithms in Proteome Discoverer 2.2 (Thermo Fisher Scientific). Raw spectrum data were searched against the UniProtKB/Swiss-Prot protein database for Zebrafish (downloaded from www.uniprot.org on 9/8/20). Search parameters included: trypsin, maximum missed cleavage site of two, precursor mass tolerance of 1.5 Da, fragment mass tolerance of 0.8 Da, fixed modification of cysteine carbamidomethylation (+ 57.021 Da), and variable modification of oxidation/hydroxylation of methionine, proline, and lysine (+ 15.995 Da). Decoy database search was performed to calculate false discovery rate (FDR). Proteins containing two or more peptides with FDR ≤ 0.01were considered positively identified.

#### Immunofluorescence

Zebrafish embryos were fixed in 4% PFA and embedded in paraffin prior to sectioning. Samples were cut into 10 µm sections. Sections were deparaffinized in Histoclear and rehydrated in a graded alcohol series. Sections were rinsed with 0.1% Tween-20 in PBS and Triton X-100 at room temperature. Blocking was performed in 5% goat serum and 2% Tween-20 in PBS for 1 h. Primary antibodies were diluted in blocking solution at 1:1000 and samples were incubated at 4 °C overnight. Secondary antibody was diluted 1:200 in blocking solution and applied to samples overnight in the dark. Prolong™ Gold antifade mountant with 4ʹ,6-diamidino-2-phenylindole (DAPI) (Fisher Scientific) was used to mount the coverslip. Samples were imaged on a Zeiss LSM 510 Meta confocal microscope.

### Results

#### Epitope selection

**Fig. 1:**
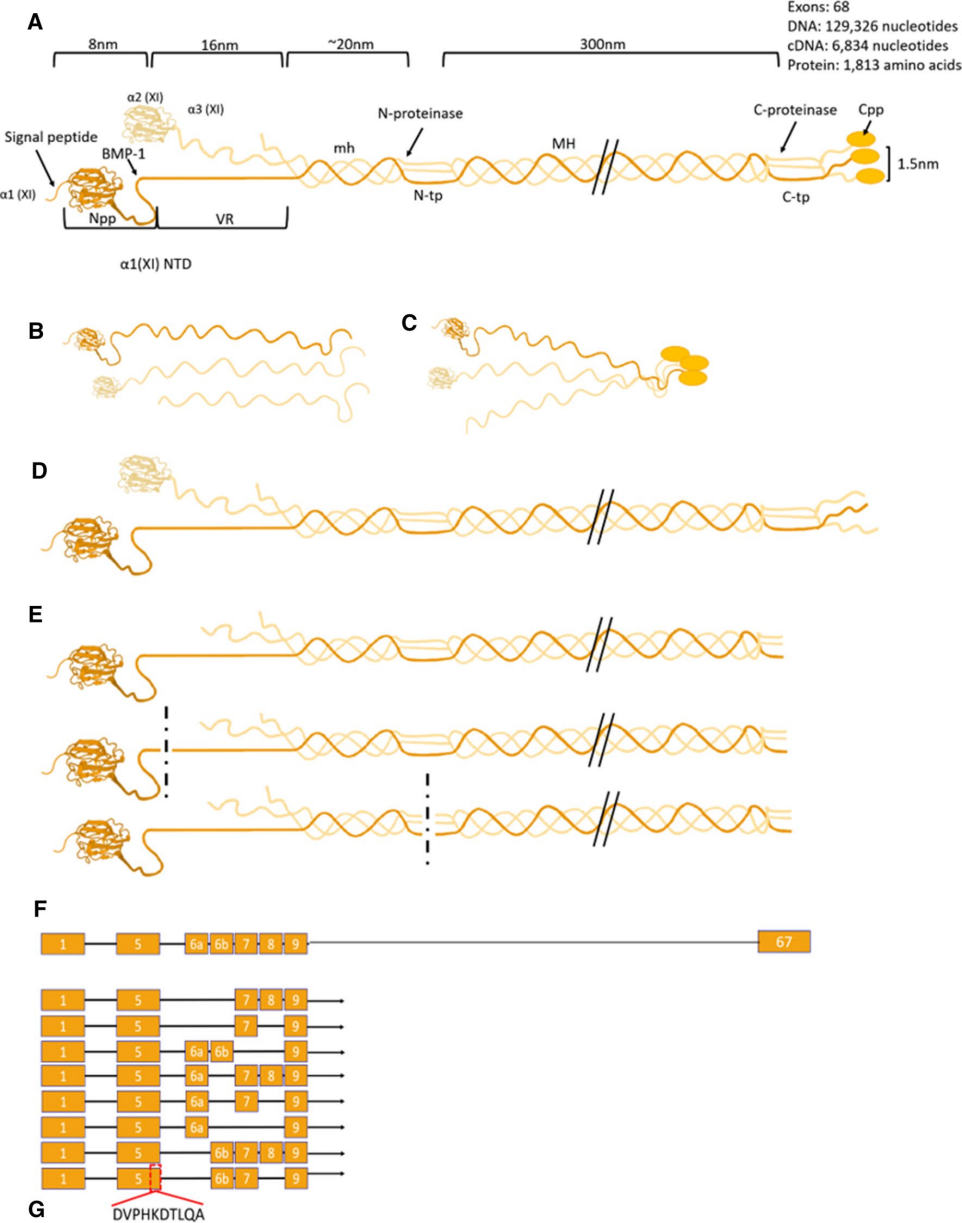
Model of Collagen type XI. **A** Collagen type XI molecule. Structural regions of the type XI collagen molecule are indicated including the signal peptide, amino terminal propeptide, variable region, the minor helix, the amino telopeptide, the major triple helix, the carboxy telopeptide, and the carboxy propeptide. Relative size and dimensions are shown above the molecular model. **B** Three alpha chains: α1(XI), α2(XI), and α3(XI). **C** Formation of triple helical molecule is initiated at the C-terminus. **D** Fully assembled triple helical collagen molecule with carboxyl propeptide. **E** Potential cleavage events, with carboxy propeptide removed, with amino propeptide removed at the BMP-1 cleavage site (35 kDa), and finally, with the ADAMTS2 cleavage site within the amino telopeptide indicated by the vertical dotted line that would release the major triple helix and generate a fragment containing the amino propeptide and variable region (100 kDa). **F** Exon structure of Col11a1a, indicating alternatively spliced isoforms. **G** Epitope sequence unique to the new antibody. This sequence is encoded by exon 5 which is present in all spliceforms. *NTD* amino terminal domain, *Npp* amino propeptide domain, *VR* variable region, *mh* minor helix, *N-tp* amino telopeptide, *MH* major triple helix, *C-tp* carboxy telopeptide, *Cpp* carboxy propeptide

We identified a unique sequence within the zebrafish collagen α1(XI) protein corresponding to the NTD [29–31]. This sequence is encoded by exon 5, preceding the start of the variable region (Fig. 1).

#### Immunoblot analysis

**Fig. 2:**
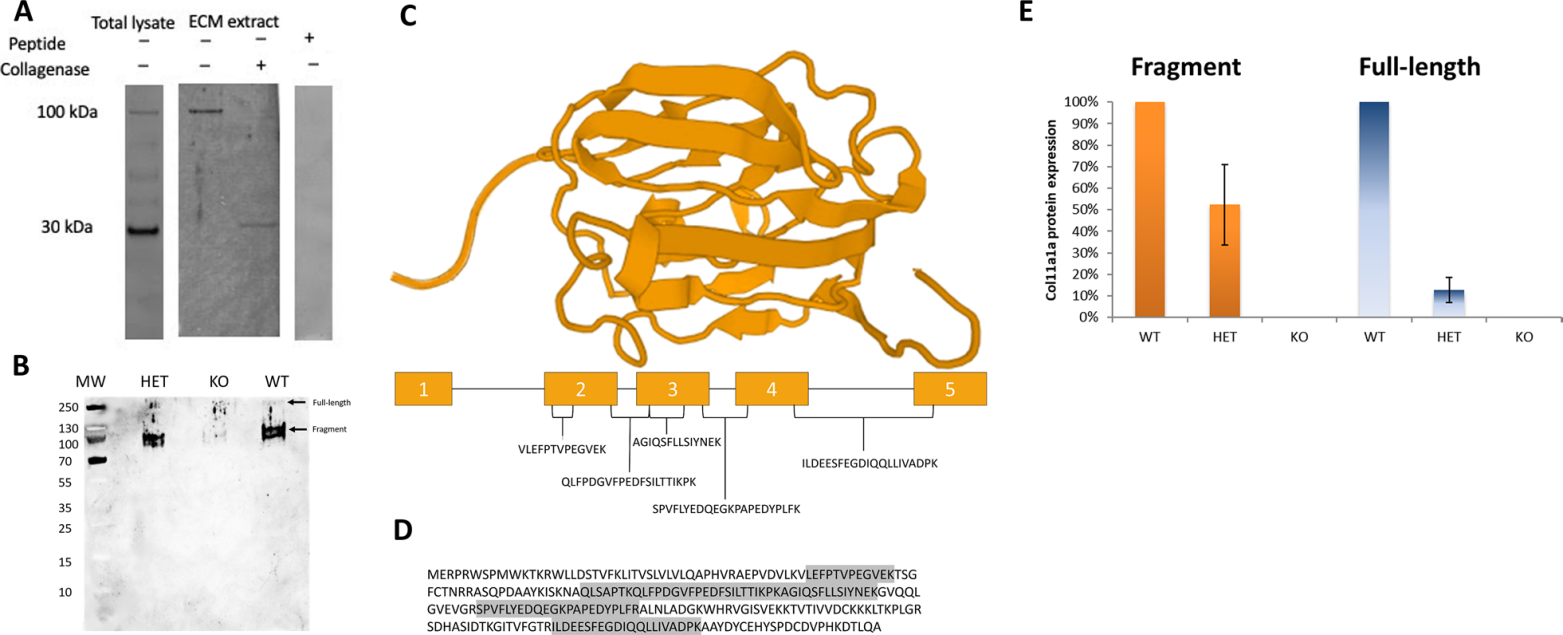
Antibody detection of Col11a1a protein by immunoblot and confirmed by mass spectrometry. **A** The antibody recognized protein bands migrating with apparent molecular weights of 100 and 35 kDa from 24 hpf zebrafish total lysate. Proteins extracted from the ECM contained the 100 kDa that was converted to a 35 kDa band upon treatment with collagenase. In the presence of a large excess of the peptide used as the antigen, the 100 kDa and 35 kDa bands from total lysate are not visible on the immunoblot, indicating competition by the peptide for the antigen binding site on the antibody. See Addiitonal file 1: Figure S1 for full length original unprocessed blots. **B** Representative immunoblot of proteins extracted from wildtype, heterozygous, and homozygous knockout zebrafish. The 100 kDa fragment of Col11a1a is apparent in lysate from 72 hpf zebrafish with quantitatively minor bands at the position of full-length Col11a1a. **C** Structural model of the amino propeptide domain of Col11a1a. Peptides detected by mass spectrometry are indicated in their respective locations of the exons encoding the protein. **D** Protein sequence of Col11a1a amino propeptide domain with grey shading indicates the sequence coverage used to confirm the identity of the protein recognized by the new antibody as Col11a1a. **E** Quantification of immunoblot band intensity from proteins extracted from wildtype (WT), heterozygotes, and homozygous knockout embryos. The NTD fragment and full-length molecule were quantified from three individual blots by densitometry, calculating average and standard deviation. Absence of these protein bands from homozygous knockout embryos confirmed specificity of the new antibody. *WT* wildtype, *HET* heterozygous, *KO* knockout, *MW* molecular weight markers

We tested the specificity of the antibody against protein extracted from whole zebrafish lysates by immunoblot. The antibody recognized protein bands with apparent molecular weights of 100 kilodaltons (kDa) and 35 kDa from total lysate collected from embryos at 24 hpf corresponding to fragments of Col11a1a without recognizing full-length Col11a1a under experimental conditions used. Collagenase treatment of an ECM extract resulted in the depletion of the 100 kDa band and a concomitant generation of a protein band with an apparent molecular weight of 35 kDa (Fig. 2A). Specificity for Col11a1a was also demonstrated by immunoblot using proteins extracted from wildtype, heterozygous ( +/−, and homozygous (−/−) knockout animals (Fig. 2B).

The antibody recognized the peptide originally used to generate the antibody under the conditions of the immunoblot, as indicated by observed competition by the peptide and abolition of the detection of protein bands at 100 kDa and 35 kDa (Fig. 2A).

#### Protein sequence analysis

The protein bands identified by immunoblot were excised and submitted for mass spectrometry to confirm identity of the proteins by sequencing (Fig. 2B and C). Mass spectrometry results of the band migrating with an apparent molecular weight of 100 kDa identified peptides from the NTD of Col11a1a. The NTD peptides were also identified in a band with an apparent molecular weight of 35 kDa (Fig. 2C and D).

#### Specificity of Col11a1a antibody

To confirm antibody specificity, we tested the antibody on proteins extracted from Col11a1a knockout zebrafish. Immunoblot indicated a lower expression of Col11a1a in heterozygous embryos and no expression in knockout homozygous embryos. Fragment (100 kDa) and very low levels of full length Col11a1a were observed in wildtype and heterozygous embryos while knockout embryos displayed no Col11a1a protein expression (Fig. 2E).

#### Immunohistochemistry

**Fig. 3:**
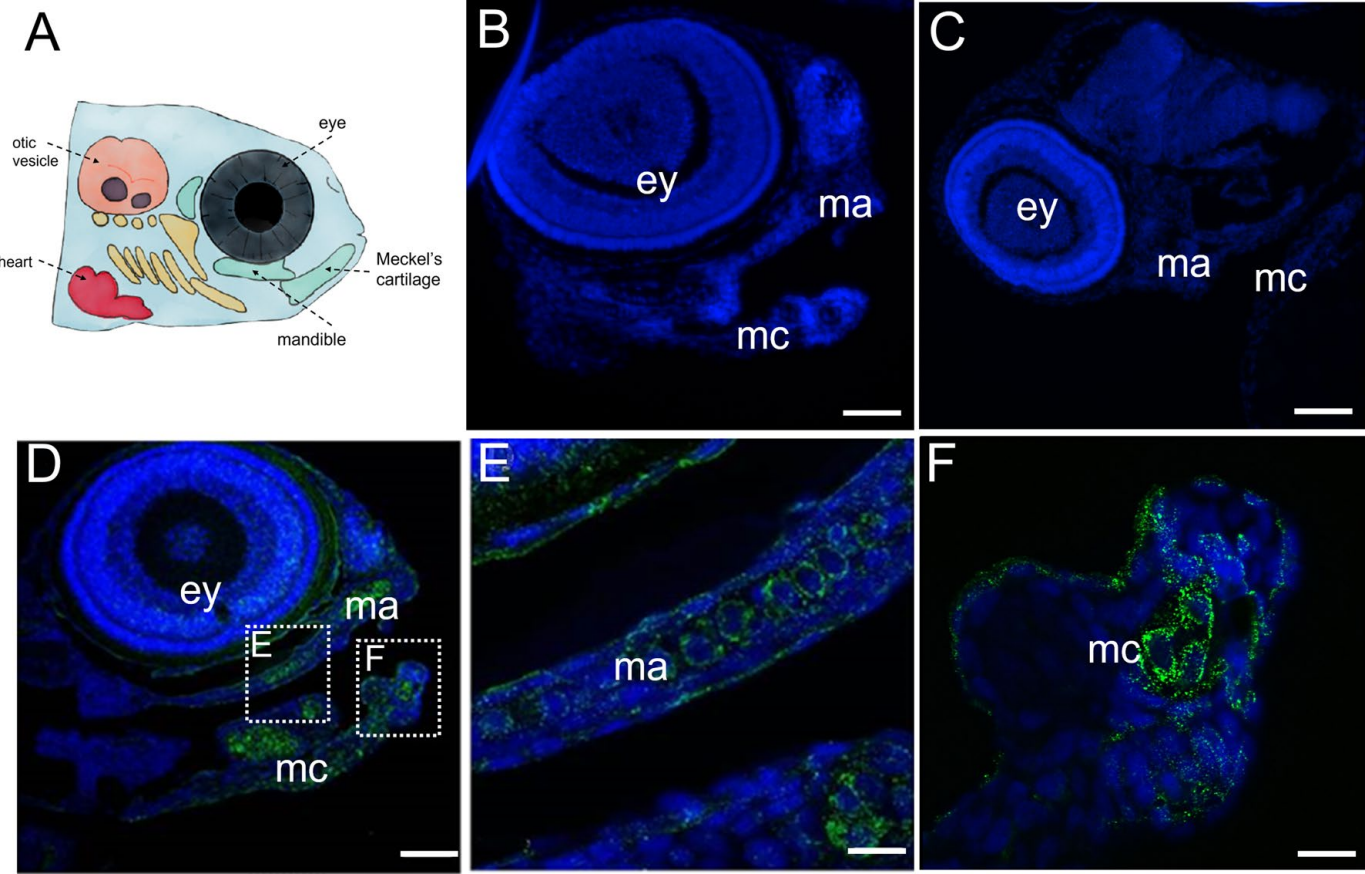
Immunohistochemistry demonstrating location of Col11a1a within developing craniofacial region at 72 hpf. Wildtype zebrafish embryos were fixed, embedded, and sectioned for immunofluorescence detection of Col11a1a using the new primary antibody. **A** Schematic representation of the craniofacial region of zebrafish. **B** Negative control using pre-immune serum in place of primary antibody. **C** Negative control omitting primary antibody. **D** Col11a1a primary antibody (green) detects Col11a1a in the eye and within the jaw cartilage elements represented by the mandible (ma) and Meckel’s cartilage (mc). DAPI (blue) staining indicates the location of cell nuclei. Dotted square frames indicate location of higher magnification images of mandibular cartilage shown in **E** and Meckel’s cartilage shown in **F**. Scale bars **B** 50 µm; **C** 50 µm; **D** 50 µm; **E** 250 µm; **F** 250 µm. Results are representative of staining pattern found in 15 individual embryos

We confirmed that the antibody recognized tissues in which expression of collagen type XI is known to occur including the mandible, Meckel’s cartilage, and eye structures at 72 hpf (Fig. 3). Specificity was demonstrated by the absence of staining in the negative controls using pre-immune serum or in the absence of primary antibody.

### Discussion

Collagens are essential for establishing tissue morphology during embryological development. Our goal was to develop an antibody recognizing Col11a1a of zebrafish. We chose an epitope that was unique to zebrafish Col11a1a within the NTD of Col11a1a that could be used for both immunoblotting and immunohistochemistry. The detection of a protein migrating with an apparent molecular weight of 100 kDa is consistent with a fragment generated by proteolytic cleavage by A Disintegrin And Metalloproteinase With Thrombospondin Motifs 2 (ADAMTS2) within the amino telopeptide [31, 32]. The recognition of the 35 kDa protein band is consistent with a released NTD fragment upon collagenase digestion to remove the minor helix or alternatively, proteolytic processing by bone morphogenetic protein-1 (BMP-1) [31, 33, 34]. Together, these results support the utility of the new antibody to recognize biologically relevant forms of Col11a1a in the zebrafish embryo.

Mass spectrometry identified peptides unique to Col11a1a in bands identified by the new antibody, supporting future investigations of collagens during development [35, 36]. Important aspects of the molecular processing of procollagens that results in mature collagens [37, 38] and the fates of fragments [39] may be revealed using this antibody. Additionally, this antibody may facilitate the localization of collagens in tissues such as skin, bones, and ligaments [40–43]. The epitope recognized by the new antibody is unique to zebrafish Col11a1a and the antibody did not recognize other collagen types. Immunoblot analysis of Col11a1a knockouts confirmed specificity of the Col11a1a antibody.

Tissue staining within the eye and craniofacial cartilage of the jaw at 72 hpf is consistent with results from other species [40]. The Col11a1 chondrodystrophic (*cho*) mouse is characterized by deficiencies in craniofacial chondrogenesis [40, 44]. Human disorders such as type 2 Stickler syndrome due to a mutation in COL11A1, display a small jaw, high myopia, and retinal detachment [18, 45–47].

Collagen type XI nucleates and limits the diameter of collagen type II fibrils and interacts with non-collagenous molecules. Previous studies demonstrated severe changes in the ECM and collagen networks when Col11a1a was knocked down in zebrafish [1, 18]. Additionally, Col11a1 mutations in the *cho* mouse show disordered chondrocytes and collagen fibrils in the growth plate [40–43].

We have generated a novel tool for monitoring changes in Col11a1a synthesis and localization in zebrafish tissue. We show that the antibody is useful for immunoblot and immunohistochemistry and confirmed the expression in cartilage of the developing skeleton as expected for collagen type XI. Future studies will rely on this antibody to investigate zebrafish models of human diseases related to COL11A1.

## Limitations

- Additional collagens migrated with apparent molecular weight (MW) of approximately 100 kDa specifically, collagen XIIα1, however, this collagen does not contain the epitope.
- Homozygous and heterozygous offspring were separated based on severity of observed phenotype. It is possible that some more severely affected heterozygotes were classified as homozygotes, leading to low levels of Col11a1a present in protein extracts from the homozygous knockout group. However, based on original homozygous embryo data before outcrossing, we feel confident in our ability to distinguish homozygous knockouts from heterozygotes.
- It is unclear how efficiently the antibody recognizes full-length Col11a1a, due low levels of protein present. Patterns of expression may change over time as the embryo matures.

## Supplementary Information

**Additional file 1: Figure S1.** Full-length lanes from unprocessed images of immunoblots. (A) Immunoblot of total lysate from zebrafish embryos with Col11a1a antibody. Lane 1—2. 24 hpf embryo protein extract, 10 μg and 5 μg total protein loaded onto gel, respectively. (B) Immunoblot of extracellular matrix extract of 24 hpf embryos, 10 μg of total protein loaded onto gel. Lane 1. Col11a1a prior to collagenase treatment (minus collagenase (coll’ase)) showing 100 kDa bond. Lane 2. Col11a1a after collagenase treatment (plus collagenase (coll’ase)) showing 35 kDa band. (C) Immunoblot demonstrating antibody specificity to Col11a1a epitope. Lane 1. Col11a1a peptide loaded onto gel and recognized by antibody. Lane 2. Sample depleted of epitope-containing proteins prior to loading onto gel for immunoblot. (D) Lane 1. 24 hpf embryo protein extract (shown in panel A), incubated in the presence of antibody plus a large excess of immunogenic peptide (shown in panel C). The peptide competes for antibody binding to the Col11a1a-related polypeptides on the blot.

## Data Availability

The datasets used and/or analyzed during the current study are available from corresponding author on request.

## Abbreviations

ADAMTS2: A disintegrin and metalloproteinase with thrombospondin motifs 2
BMP-1: Bone morphogenetic protein -1
cho: Chondrodystrophic
DAPI: 4ʹ,6-Diamidino-2-phenylindole
ECM: Extracellular matrix
ELISA: Enzyme-linked immunosorbent assay
FDR: False discovery rate
Hpf: Hours post-fertilization
IACUC: Institutional Animal Care and Use Committee
kDa: Kilodaltons
LC–MS/MS: Liquid chromatography tandem mass spectrometry
MW: Molecular weight
NTD: Amino terminal domain
PFA: Paraformaldehyde
SDS: Sodium dodecyl sulfate
TTBS: Tween-20 Tris buffered saline
