# Supplementary material for: Authentication of a novel antibody to zebrafish collagen type XI alpha 1 chain (Col11a1a)
Source: BMC Res Notes. 2021 Sep 15;14:359. doi: 10.1186/s13104-021-05770-x (PMC8444443; doi:10.1186/s13104-021-05770-x)
Supplement: Supplementary file 1 — Additional file 1: Figure S1. Full-length lanes from unprocessed images of immunoblots. (A) Immunoblot of total lysate from zebrafish embryos with Col11a1a antibody. Lane 1—2. 24 hpf embryo protein extract, 10 μg and 5 μg total protein loaded onto gel, respectively. (B) Immunoblot of extracellular matrix extract of 24 hpf embryos, 10 μg of total protein loaded onto gel. Lane 1. Col11a1a prior to collagenase treatment (minus collagenase (coll’ase)) showing 100 kDa bond. Lane 2. Col11a1a after collagenase treatment (plus collagenase (coll’ase)) showing 35 kDa band. (C) Immunoblot demonstrating antibody specificity to Col11a1a epitope. Lane 1. Col11a1a peptide loaded onto gel and recognized by antibody. Lane 2. Sample depleted of epitope-containing proteins prior to loading onto gel for immunoblot. (D) Lane 1. 24 hpf embryo protein extract (shown in panel A), incubated in the presence of antibody plus a large excess of immunogenic peptide (shown in panel C). The peptide competes for antibody binding to the Col11a1a-related polypeptides on the blot. [file 13104_2021_5770_MOESM1_ESM.pdf]

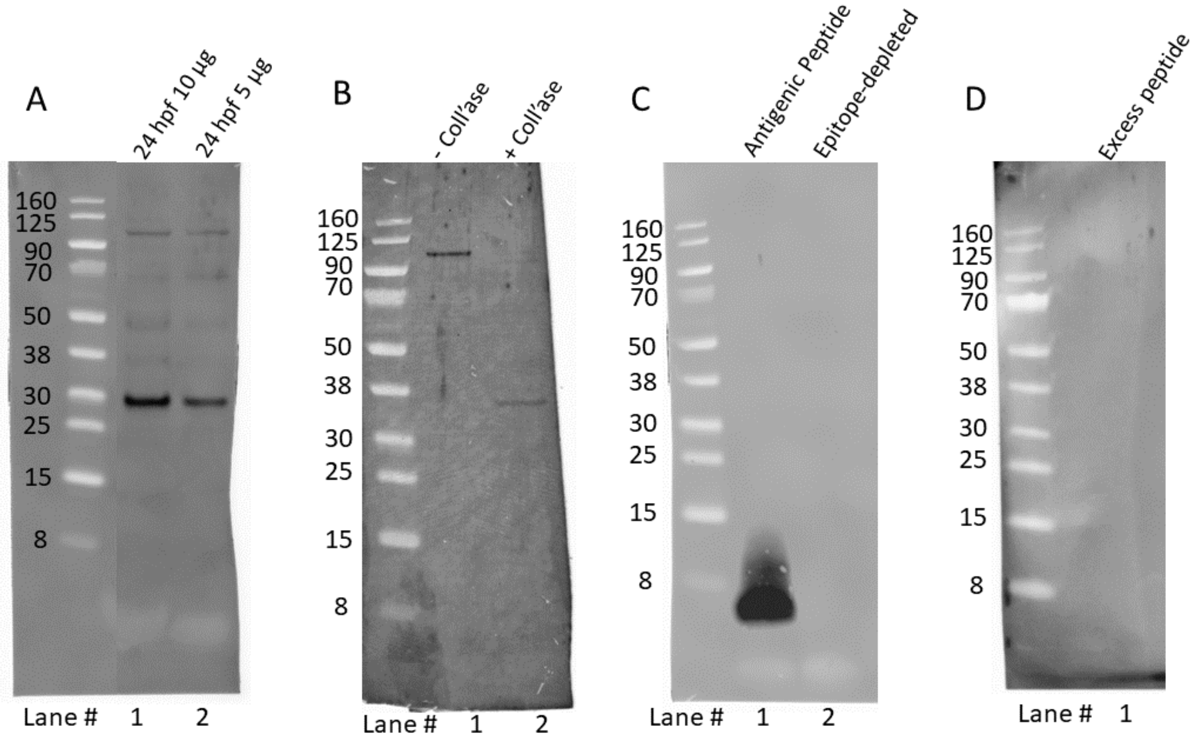

Supplementary Figure 1. Full-length lanes from unprocessed images of immunoblots. (A) Immunoblot of total lysate from zebrafish embryos with Col11a1a antibody. Lane 1 – 2. 24 hpf embryo protein extract, 10 µg and 5 µg total protein loaded onto gel, respectively. (B) Immunoblot of extracellular matrix extract of 24 hpf embryos, 10 µg of total protein loaded onto gel. Lane 1. Col11a1a prior to collagenase treatment (minus collagenase (coll'ase)) showing 100 kDa bond. Lane 2. Col11a1a after collagenase treatment (plus collagenase (coll'ase)) showing 35 kDa band. (C) Immunoblot demonstrating antibody specificity to Col11a1a epitope. Lane 1. Col11a1a peptide loaded onto gel and recognized by antibody. Lane 2. Sample depleted of epitope-containing proteins prior to loading onto gel for immunoblot. (D) Lane 1. 24 hpf embryo protein extract (shown in panel A), incubated in the presence of antibody plus a large excess of immunogenic peptide (shown in panel C). The peptide competes for antibody binding to the Col11a1a-related polypeptides on the blot.
